# Risk of pulmonary embolism and deep vein thrombosis following COVID‐19: a nationwide cohort study

**DOI:** 10.1002/mco2.655

**Published:** 2024-07-14

**Authors:** Hye Jun Kim, Seogsong Jeong, Jihun Song, Sun Jae Park, Young Jun Park, Yun Hwan Oh, Jaehun Jung, Sang Min Park

**Affiliations:** ^1^ Department of Biomedical Sciences Seoul National University College of Medicine Seoul South Korea; ^2^ Department of Biomedical Informatics Korea University College of Medicine Seoul South Korea; ^3^ Medical Research Center Genomic Medicine Institute Seoul National University Seoul South Korea; ^4^ Department of Family Medicine Chung‐Ang University Gwangmyeong Hospital Chung‐Ang University College of Medicine Gwangmyeong South Korea; ^5^ Department of Preventive Medicine Gachon University College of Medicine Incheon South Korea; ^6^ Department of Family Medicine Seoul National University Hospital Seoul South Korea

**Keywords:** embolism, long‐term sequelae, postacute COVID‐19 syndrome, SARS‐CoV‐2, thrombosis

## Abstract

Recent studies elucidate that coronavirus disease 2019 (COVID‐19) patients may face a higher risk of cardiovascular complications. This study aimed to evaluate association of COVID‐19 with the risk of pulmonary embolism (PE) or deep vein thrombosis (DVT). This nationwide population‐based retrospective cohort study included Korean adult citizens between January 2021 and March 2022 from the Korea Disease Control and Prevention Agency COVID‐19 National Health Insurance Service cohort. The Fine and Gray's regression with all‐cause death as a competing event was adopted to evaluate PE and DVT risks after COVID‐19. This study included a total of 1,601,835 COVID‐19 patients and 14,011,285 matched individuals without COVID‐19. The risk of PE (adjusted hazard ratio [aHR], 6.25; 95% confidence interval [CI], 3.67–10.66; *p *< 0.001) and DVT (aHR, 3.05; 95% CI, 1.75–5.29; *p *< 0.001) was higher in COVID‐19 group in individuals without complete COVID‐19 vaccination. In addition, individuals with complete COVID‐19 vaccination still had a higher risk of COVID‐19‐related PE (aHR, 1.48; 95% CI, 1.15–1.88; *p *< 0.001). However, COVID‐19 was not a significant risk factor for DVT among those with complete COVID‐19 vaccination. COVID‐19 was identified as an independent factor that elevated PE and DVT risks, especially for individuals without complete COVID‐19 vaccination.

## Graphical abstract

COVID‐19 was associated with an increased risk of PE (aHR, 6.25; 95% CI, 3.67–10.66; *p *< 0.001) and DVT (aHR, 3.05; 95% CI, 1.75–5.29; *p *< 0.001) in unvaccinated participants. Even in those with complete COVID‐19 vaccination, a smaller but significant risk of PE was observed (aHR, 1.48; 95% CI, 1.15–1.88; *p *< 0.001). However, COVID‐19 did not increase DVT risk in those with complete vaccination.

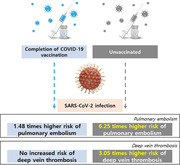

## 1 INTRODUCTION

Until the end of 2023, the World Health Organization had reported 773.8 million cases of coronavirus disease 2019 (COVID‐19), an infectious disease caused by severe acute respiratory syndrome coronavirus 2 (SARS‐CoV‐2), along with 7.0 million deaths.^1^ Recent studies have reported that those with COVID‐19 may be at a higher risk of late clinical sequelae including heart disease and cardiovascular complications.^2^, ^3^, ^4^ However, there is insufficient evidence regarding pulmonary embolism (PE) and deep vein thrombosis (DVT) as clinical manifestation of COVID‐19. A recent meta‐analysis has shown a higher risk of PE and DVT, with cumulative incidences of 1.2 and 2.3%, respectively.^5^, ^6^ However, this study has pooled results from hospital admission‐based cases, and such high incidences may not accurately reflect the general population in real‐world settings.

COVID‐19 vaccination has been associated with a reduced risk of cardiovascular diseases, including ischemic stroke and acute myocardial infarction, after SARS‐CoV‐2 infection.^7^ A recent study of thrombosis and thrombocytopenia after COVID‐19 vaccination found that the second dose of BNT162b2 vaccination is protective against DVT, whereas the first dose of BNT162b2 vaccination increased the risk of PE.^8^ In addition, both PE and DVT risk increased dramatically after COVID‐19. In contrast, another study found that at least one vaccination dose is protective against PE and DVT.^9^

In a study from Sweden, COVID‐19 has been recognized as a risk factor for the onset of DVT, PE, and bleeding.^10^ The researchers evaluated the incidence rate ratio and risk ratio after adjusting for a limited set of covariates that lacked some important information, such as COVID‐19 vaccination status and individual‐level health screening examination results.^10^ In addition, another study from Sweden found that hospitalized patients with COVID‐19 are at a higher risk of venous thromboembolism.^11^ However, this study did not assess the risk among non‐hospitalized patients with COVID‐19 and did not take into account COVID‐19 vaccination status.^11^ In contrast, another study reported a high prevalence of PE in non‐hospitalized patients with mild COVID‐19.^12^ Likewise, previous studies were limited in terms of determination of the interaction between COVID‐19 and COVID‐19 vaccination, the evaluation period that only enrolled COVID‐19 patients up to 2021, the study participants mostly consisted of Western populations despite a previous study suggesting race may be related to PE risk after COVID‐19,^13^ and a lack of consideration for important potential covariates, such as COVID‐19 vaccinations and health screening examination results.

Therefore, this study sought to determine the effects of COVID‐19 on PE or DVT risk in South Korean population, who faced an elevated risk of COVID‐19 transmission due to high population density despite strict social distancing and self‐isolation measures, after separating the effects of COVID‐19 itself from the vaccinations, confirming the interaction between the risks of COVID‐19 and COVID‐19 vaccinations, extensively adjusting for potential confounders, and enlarging the study population to include a more recent phase. In addition, the association between COVID‐19 severity and PE or DVT risk based on COVID‐19 vaccination strata was evaluated in a South Korean nationwide population‐based cohort.

## 2 RESULTS

Table 1 provides an overview of the descriptive characteristics of the individuals participating in the present study. Out of a total of 15,613,120 participants, 14,011,285 were without COVID‐19 and 1,601,835 were with COVID‐19. Participants diagnosed with COVID‐19 tended to be man, possess a higher household income, have diabetes, dyslipidemia, exhibit fewer comorbidities, present a higher body mass index, less likely to smoke, drink, engage in physical exercise, and more likely to be vaccinated.

**TABLE 1:** Descriptive characteristics of the South Korean participants with and without COVID‐19.

	No COVID‐19 (*n* = 14,011,285)	COVID‐19 (*n* = 1,601,835)	*p* Value
Age, years	47.6 (13.8)	47.6 (13.9)	0.012
Sex, *n* (%)			<0.001
Men	8,486,437 (60.6)	973,095 (60.8)	
Women	5,524,848 (39.4)	628,740 (39.2)	
Household income,^a^ *n* (%)			<0.001
1st quartile (lowest)	4,460,138 (31.8)	504,582 (31.5)	
2nd quartile	3,778,288 (27.0)	432,957 (27.0)	
3rd quartile	3,088,705 (22.0)	351,796 (22.0)	
4th quartile (highest)	2,684,154 (19.2)	312,500 (19.5)	
Hypertension, *n* (%)	2,412,873 (17.2)	275,699 (17.2)	<0.001
Diabetes, *n* (%)	960,045 (6.9)	104,104 (6.5)	<0.001
Dyslipidemia, *n* (%)	864,794 (6.2)	101,165 (6.3)	<0.001
Charlson comorbidity index, *n* (%)			<0.001
0	4,681,354 (33.4)	570,836 (35.6)	
1	6,648,112 (47.5)	728,321 (45.5)	
≥2	2,681,819 (19.1)	302,678 (18.9)	
Body mass index (kg/m^2^)	24.6 (4.3)	24.7 (3.6)	<0.001
Waist circumference (cm)	83.1 (10.9)	83.5 (11.1)	<0.001
Systolic blood pressure (mmHg)	125.9 (14.8)	125.8 (14.6)	0.002
Diastolic blood pressure (mmHg)	78.7 (10.3)	78.7 (10.3)	0.975
Total cholesterol (mg/dL)	198.6 (40.6)	198.4 (39.8)	<0.001
Creatinine	0.9 (0.5)	0.9 (0.5)	<0.001
Hemoglobin	14.7 (1.5)	14.7 (1.5)	<0.001
Fasting blood glucose	104.8 (26.7)	104.2 (25.0)	<0.001
Cigarette smoking, *n* (%)			<0.001
Nonsmoker	7,098,872 (50.7)	866,168 (54.1)	
Past smoker	4,616,722 (32.9)	537,531 (33.5)	
Current smoker	2,295,691 (16.4)	198,136 (12.4)	
Alcohol consumption, *n* (%)			<0.001
Yes	5,654,601 (40.4)	643,488 (40.2)	
No	8,356,684 (59.6)	958,347 (59.8)	
MVPA, *n* (%)			<0.001
0 time/week	3,142,423 (22.4)	324,874 (20.3)	
1–2 time/week	2,393,955 (17.1)	273,789 (17.1)	
3–4 time/week	2,746,535 (19.6)	322,301 (20.1)	
≥5 time/week	5,728,372 (40.9)	680,871 (42.5)	
COVID‐19 vaccination, *n* (%)			<0.001
Completion of the primary series	13,058,901 (93.2)	1,515,239 (94.6)	
Unvaccinated	952,384 (6.8)	86,596 (5.4)	
COVID‐19 phase, *n* (%)			<0.001
2021/01/01–2021/06/30	126,639 (0.9)	13,422 (0.8)	
2021/07/01–2022/01/15	626,184 (4.5)	63,383 (4.0)	
2022/01/16–2022/03/31	13,258,462 (94.6)	1,525,030 (95.2)	
COVID‐19 severity, *n* (%)			–
Mild‐low risk	–	924,366 (57.7)	
Mild‐high risk	–	655,369 (40.9)	
Serious	–	20,357 (1.3)	
Severely serious	–	1490 (0.1)	

Variables are presented as mean (standard deviation) if normally distributed, unless indicated otherwise.

Abbreviations: COVID‐19, coronavirus disease; MVPA, moderate‐to‐vigorous physical activity.

^a^ Proxy for socioeconomic status.

After full adjustment for covariates, it was observed that SARS‐CoV‐2 infection is independently associated with an elevated PE risk in unvaccinated participants (adjusted hazard ratio [aHR], 6.25; 95% confidence interval [CI], 3.67–10.66). Additionally, individuals who had a complete COVID‐19 vaccination also exhibited a slightly increased risk of COVID‐19‐related PE (aHR, 1.48; 95% CI, 1.15–1.88), although the magnitude of this effect was notably smaller compared with the unvaccinated group (Table 2). Moreover, the risks of PE (aHR, 1.31; 95% CI, 0.97–1.75; *p *= 0.075) and DVT (aHR, 1.16; 95% CI, 0.93–1.44; *p *= 0.190) by COVID‐19 were attenuated to statistically not significant levels among participants with 3rd dose or more vaccinations. (Tables S1 and S2). In addition, COVID‐19 in the pre‐omicron phase was associated with an elevated risk of PE regardless of the status of COVID‐19 vaccination (Table S3). However, a significant interaction was found for the effect of COVID‐19 in the omicron phase with COVID‐19 vaccination status (*p* for interaction = 0.003), indicating that those with completion of the primary series had a lower risk of PE due to COVID‐19 as compared with unvaccinated participants (Table S4). Regarding DVT, SARS‐CoV‐2 infection was recognized as an independent risk factor for incident DVT among unvaccinated participants (aHR, 3.05; 95% CI, 1.75–5.29), whereas no significant association between COVID‐19 and DVT was observed among those with complete COVID‐19 vaccination (aHR, 1.15; 95% CI, 0.94–1.40; Table 3). As for COVID‐19 in the pre‐omicron phase, no significant association was found between COVID‐19 and DVT, possibly due to a limited number of cases (Table S5). However, DVT risk was significantly higher among participants with COVID‐19 in the omicron phase and without prior COVID‐19 vaccinations, whereas the risk of DVT was not elevated among participants with completion of the primary series (Table S6).

**TABLE 2:** Association of COVID‐19 with pulmonary embolism.

	No COVID‐19 (*n* = 14,011,285)	COVID‐19 (*n* = 1,601,835)	*p* Value	*p* for interaction
Unvaccinated				
Study population, *n*	952,384	86,596		
Event, *n* (%)	36 (0.00)	22 (0.03)		
Person‐years	503,268	49,441		
Median time‐to‐event, days	90 (44‐194)	36 (26‐86)		
HR (95% CI)	1.00 (reference)	6.75 (3.97–11.47)	<0.001	<0.001
aHR (95% CI)^a^	1.00 (reference)	6.25 (3.67–10.65)	<0.001	<0.001
aHR (95% CI)^b^	1.00 (reference)	6.25 (3.67–10.66)	<0.001	<0.001
Completion of the primary series				
Study population, *n*	13,058,901	1,515,239		
Event, *n* (%)	435 (0.00)	74 (0.00)		
Person‐years	4,046,980	465,882		
Median time‐to‐event, days	52 (27‐82)	46 (27–78)		
HR (95% CI)	1.00 (reference)	1.47 (1.15–1.88)	0.002	
aHR (95% CI)^a^	1.00 (reference)	1.48 (1.15–1.89)	0.002	
aHR (95% CI)^b^	1.00 (reference)	1.48 (1.15–1.88)	0.002	

HRs were evaluated using the Cox proportional hazards regression.

Abbreviations: COVID‐19, coronavirus disease; HR, hazard ratio; CI, confidence interval; aHR, adjusted hazard ratio.

^a^ Adjusted for age, sex, household income, Charlson comorbidity index, hypertension, diabetes, dyslipidemia, body mass index, moderate‐to‐vigorous physical activity, smoking, and alcohol consumption.

^b^ Assessed using the Fine and Gray's regression with death as a competing risk to calculate subdistribution hazard ratios after adjustments for variables that are included in the model A.

**TABLE 3:** Association of COVID‐19 with deep vein thrombosis.

	No COVID‐19 (*n* = 14,011,285)	COVID‐19 (*n* = 1,601,835)	*p* Value	*p* for interaction
Unvaccinated				
Study population, *n*	952,384	86,596		
Event, *n* (%)	56 (0.01)	16 (0.02)		
Person‐years	503,247	49,439		
Median time‐to‐event, days	105 (46–206)	55 (33–203)		
HR (95% CI)	1.00 (reference)	3.16 (1.81–5.50)	<0.001	0.001
aHR (95% CI)^a^	1.00 (reference)	3.05 (1.75–5.31)	<0.001	0.001
aHR (95% CI)^b^	1.00 (reference)	3.05 (1.75–5.29)	<0.001	0.001
Completion of the primary series				
Study population, *n*	13,058,901	1,515,239		
Event, *n* (%)	848 (0.01)	111 (0.01)		
Person‐years	4,046,899	465,878		
Median time‐to‐event, days	61 (32–89)	59 (28–82)		
HR (95% CI)	1.00 (reference)	1.13 (0.93–1.38)	0.231	
aHR (95% CI)^a^	1.00 (reference)	1.15 (0.94–1.40)	0.170	
aHR (95% CI)^b^	1.00 (reference)	1.15 (0.94–1.40)	0.170	

HRs were evaluated using the Cox proportional hazards regression.

Abbreviations: COVID‐19, coronavirus disease; HR, hazard ratio; CI, confidence interval; aHR, adjusted hazard ratio.

^a^ Adjusted for age, sex, household income, Charlson comorbidity index, hypertension, diabetes, dyslipidemia, body mass index, moderate‐to‐vigorous physical activity, smoking, and alcohol consumption.

^b^ Assessed using the Fine and Gray's regression with death as a competing risk to calculate subdistribution hazard ratios after adjustments for variables that are included in the model A.

**FIGURE 1:**
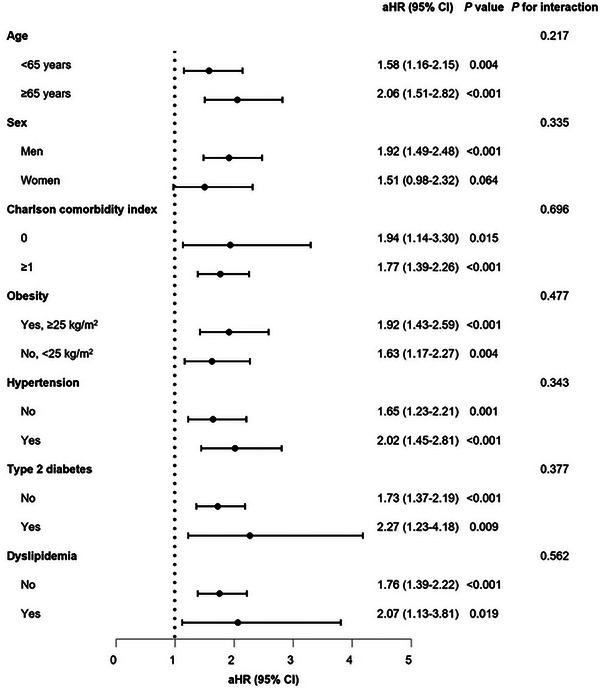
Stratified analyses on association of COVID‐19 with pulmonary embolism. aHRs were estimated using multivariable Cox proportional hazards regression after adjusting for age, sex, household income, Charlson comorbidity index, body mass index, hypertension, diabetes, dyslipidemia, moderate‐to‐vigorous physical activity, smoking, alcohol consumption, and COVID‐19 vaccination. Abbreviations: COVID‐19, coronavirus disease; aHR, adjusted hazard ratio; CI, confidence interval.

**FIGURE 2:**
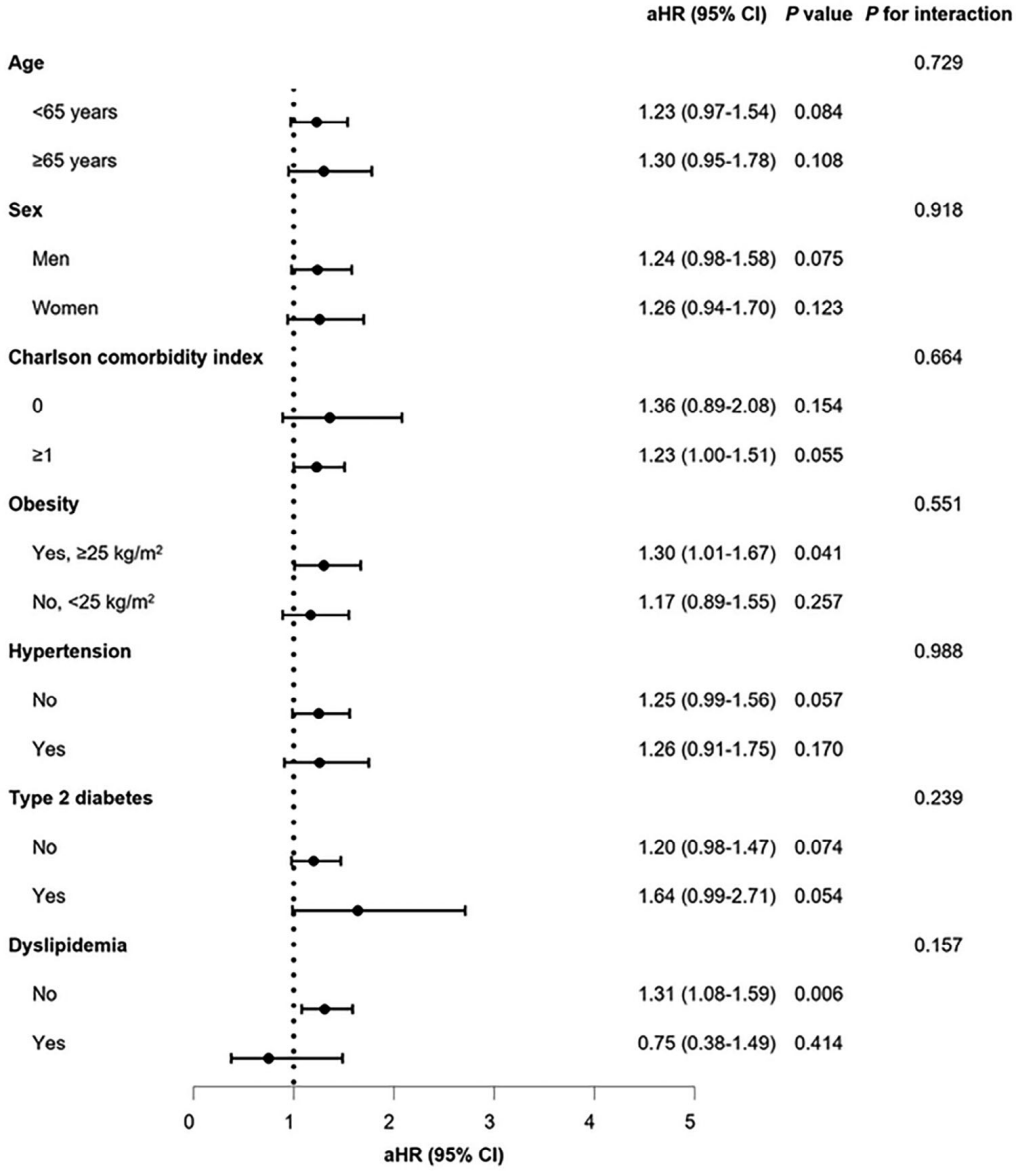
Stratified analyses on association of COVID‐19 with deep vein thrombosis. aHRs were estimated using multivariable Cox proportional hazards regression after adjusting for age, sex, household income, Charlson comorbidity index, body mass index, hypertension, diabetes, dyslipidemia, moderate‐to‐vigorous physical activity, smoking, alcohol consumption, and COVID‐19 vaccination. Abbreviations: COVID‐19, coronavirus disease; aHR, adjusted hazard ratio; CI, confidence interval.

Stratified analyses, considering age, sex, CCI, obesity, hypertension, diabetes, and dyslipidemia, revealed no noteworthy interactions for either PE and DVT (Figures 1 and 2). Upon stratifying the patients based on the severity of COVID‐19, it was noted that the risk of incident PE increased as the severity of COVID‐19 increased, regardless of vaccination status (unvaccinated: aHR, 17.20; 95% CI, 8.20–35.97; completion of the primary series: aHR, 15.15; 95% CI, 8.70–26.38). However, patients with a complete COVID‐19 vaccination experienced a comparatively reduced level of risk (Table S7). In terms of incident DVT, there was no independent association observed with the severity of COVID‐19, presumably due to the small number of cases (Table S8).

Last, when analyzing the participants based on the types of vaccine they received for the first and second dose, both receiving mRNA vaccines (aHR, 0.65; 95% CI, 0.46–0.93) and viral vector vaccines (aHR, 0.60; 95% CI, 0.41–0.86) for the first and second dose were associated with a reduced risk of developing PE after COVID‐19, as compared with those who were unvaccinated among participants without COVID‐19 (Table S9). Among patients with COVID‐19, these findings were even more significant, where receiving mRNA vaccines (aHR. 0.12; 95% CI, 0.07–0.20) and viral vector vaccines (aHR, 0.17; 95% CI, 0.10–0.29) for the first and second dose were associated with a lower PE risk after COVID‐19, as compared with the unvaccinated individuals (Table 4). In terms of DVT, both receiving mRNA vaccines (aHR, 0.33; 95% CI, 0.19–0.56) and viral vector vaccines (aHR, 0.31; 95% CI, 0.18–0.56) for the first and second dose were associated with a lower DVT risk after COVID‐19, as compared with the unvaccinated participants after COVID‐19 (Tables 5 and S10).

**TABLE 4:** Association of COVID‐19 vaccination with incident pulmonary embolism among participants with COVID‐19.

	Unvaccinated	Completion of the primary series	First and second dose vaccination	
			mRNA–mRNA	Viral–viral
Study population, *n*	86,596	1,515,239	986,482	373,966
Event, *n* (%)	22 (0.03)	74 (0.00)	32 (0.00)	40 (0.01)
Person‐years	49,441		301,841	116,495
aHR (95% CI)^a^	1.00 (reference)	0.14 (0.09–0.22)^***^	0.12 (0.07–0.20)^***^	0.17 (0.10–0.29)^***^
aHR (95% CI)^a^			1.00 (reference)	1.46 (0.91–2.34)

aHRs were estimated using the Cox proportional hazards regression.

mRNA vaccine includes Pfizer‐BioNTech (BNT162b2) and Moderna (mRNA‐1273) vaccine.

Abbreviations: COVID‐19, coronavirus disease; mRNA–mRNA, administration of mRNA vaccine for the 1st and 2nd dose of COVID‐19 vaccine; viral–viral, administration of viral vector vaccine for the 1st and 2nd dose of COVID‐19 vaccine; aHR, adjusted hazard ratio; CI, confidence interval.

^a^ Adjusted for age, sex, household income, Charlson comorbidity index, hypertension, diabetes, dyslipidemia, body mass index, moderate‐to‐vigorous physical activity, smoking, and alcohol consumption.

Viral vector vaccine includes AstraZeneca (ChAdOx1 nCov‐19) and Janssen (AD26.COV2‐S) vaccine.

*** *p* value < 0.001.

**TABLE 5:** Association of COVID‐19 vaccination with incident deep vein thrombosis among participants with COVID‐19.

	Unvaccinated	Completion of the primary series	First and second dose vaccination	
			mRNA–mRNA	Viral–viral
Study population, *n*	86,596	1,515,239	986,482	373,966
Event, *n* (%)	16 (0.02)	111 (0.01)	61 (0.01)	45 (0.01)
Person‐years	49,439		301,836	116,497
aHR (95% CI)^a^	1.00 (reference)	0.32 (0.19–0.54)^***^	0.33 (0.19–0.56)^***^	0.31 (0.18–0.56)^***^
aHR (95% CI)^a^			1.00 (reference)	0.96 (0.64–1.43)

aHRs were estimated using the Cox proportional hazards regression.

mRNA vaccine includes Pfizer‐BioNTech (BNT162b2) and Moderna (mRNA‐1273) vaccine.

Viral vector vaccine includes AstraZeneca (ChAdOx1 nCov‐19) and Janssen (AD26.COV2‐S) vaccine.

Abbreviations: COVID‐19, coronavirus disease; mRNA–mRNA, administration of mRNA vaccine for the 1st and 2nd dose of COVID‐19 vaccine; Viral–viral, administration of viral vector vaccine for the 1st and 2nd dose of COVID‐19 vaccine; aHR, adjusted hazard ratio; CI, confidence interval.

^a^ Adjusted for age, sex, household income, Charlson comorbidity index, hypertension, diabetes, dyslipidemia, body mass index, moderate‐to‐vigorous physical activity, smoking, and alcohol consumption.

*** *p* value < 0.001.

## 3 DISCUSSION

This nationwide cohort study discovered that SARS‐CoV‐2 infection may lead to an elevated risk of PE, and this association was particularly prominent in the unvaccinated participants who had not received a complete COVID‐19 vaccination. Additionally, SARS‐CoV‐2 infection was identified as an independent risk factor for DVT, although this association was not significant among individuals who had completed the primary series of COVID‐19 vaccination.

Initially tackling immediate consequences of COVID‐19, concerns then encompassed potential long‐term effects, such as pulmonary, cardiovascular, and neurological manifestations.^14^, ^15^, ^16^, ^17^ The long‐term consequences are thought to be associated with an imbalanced immune response against SARS‐CoV‐2 infection‐related cytokine storm that induces systemic inflammation and multiorgan damages.^18^ Prolonged inflammation may induce tissue damage and result in scarring, participating in the onset of complications.^19^ Furthermore, endothelial dysfunction, marked by damage to the blood vessel lining, may lead to the formation of blood clots and complications in the vascular system, which may be related to a higher risk of PE and DVT after COVID‐19, as shown in the present study.^18^, ^19^

During the earlier COVID‐19 pandemic stages, coagulopathy was considered a major target treatment for COVID‐19‐related mortality.^20^ Tang et al.^21^ indicated that anticoagulant treatment with low molecular weight heparin lowered mortality in severe COVID‐19 patients. Subsequently, some physicians and researchers suggested that anticoagulant therapy may improve the prognosis of patients with COVID‐19 who are at a higher risk of venous thromboembolism.^22^ A recent study of preventive anticoagulation upon hospital discharge found that prolonged preventive anticoagulation lowered the risk of thrombotic events without a corresponding increase in the risk of bleeding.^23^ However, conflicting results regarding the risk of thromboembolism are being reported, and the consensus regarding which participants to be involved in preventive anticoagulation is scant. Stevens et al.^24^ demonstrated that the risk of COVID‐19‐related thromboembolism is lower among ambulatory patients, but the risk was not lower among hospitalized patients who had COVID‐19 vaccination.

The risk of COVID‐19 vaccination against thromboembolism has been reported with heterogeneous results. A recent US study showed no difference in venous thromboembolism rate when compared 90 days before and after COVID‐19 vaccination.^25^ Another recent study from the United States demonstrated that one case of mRNA vaccination was considered vaccine‐induced immune thrombotic thrombocytopenia within 4–28 days after the vaccination, from a total of 74 thrombosis cases.^26^ In contrast, a recent self‐controlled cases series study reported that COVID‐19 vaccination increased the risk of thrombocytopenic, hemorrhagic, and thromboembolic events.^27^ In addition, Chui et al.^28^ found that the incidence of thromboembolic events within 28 days was lower after COVID‐19 vaccination compared with no vaccination in COVID‐19 patients. Therefore, whether COVID‐19 vaccination may be an important public health intervention against PE and DVT for individuals at a higher risk of COVID‐19 remains controversial.

The mechanism between COVID‐19 and thromboembolism may be due to the interactions of the spike glycoproteins of SARS‐CoV‐2 and platelets.^29^ Similar to heparin‐induced thrombocytopenia, the spike glycoprotein, with its high affinity to the angiotensin‐converting enzyme 2 (ACE2) receptor, has the potential to cause thrombosis via being a target that is encoded by the viral vector and mRNA vaccines.^30^ This elucidation is similar to the mechanism of thrombocytopenic thrombosis in Vaxzevria.^31^ Additionally, it has been suggested that phosphatidylserine, tissue factors, and neutrophil extracellular traps stimulate blood coagulation activity in COVID‐19 patients via various cytokine storms.^32^ After the simulated cytokine storms, neutrophils are activated, which results in the development of thrombin that activates protease activated receptor 1 for platelets to become thrombogenic.^33^ Platelets and megakaryocytes can also be infected by SARS‐CoV‐2 through binding to ACE2 on the cellular membrane, and spike protein‐ACE2 binding contributes to releasement of the dense granules’ contents, such as von Willebrand factor, serotonin, adenosine diphosphate, and platelet factor 4, that promote aggregation of platelets and thrombus formation.^29^, ^33^, ^34^ In addition, platelet factor 4 has been found to upregulate the expression of anti‐PF4/polyanion antibody that attaches to platelets, macrophage, and neutrophils by binding to the *Fcγ* receptor IIA to facilitate the interaction between cells.^35^

The major strengths of this study may be the robust control of prior thrombotic events and reduced bias through the exact matching. In addition, vaccination status‐stratified analyses were performed to separate the effects of COVID‐19 and COVID‐19 vaccination against PE and DVT. However, this study is subject to some limitations. We could not confirm the presence of preventive anticoagulation therapy. Within the enrollment period of this study, preventive anticoagulation therapy was not common for COVID‐19 patients, and we suppose there may not be a sufficiently effective number of preventive anticoagulation cases. In addition, since the SARS‐CoV‐2 infection data provided by the Korea Disease Control and Prevention Agency (KDCA) is available only until March 31, 2022, it was not possible to account for infections that may have occurred after that period. Last, the study population is not limited to a PE‐ or DVT‐high‐risk population, considering the hypothesis of this study that was to determine associations of COVID‐19 with PE and DVT risks in a real‐world population.

This study provided evidence supporting the increased risk of PE and DVT associated with COVID‐19, particularly among unvaccinated individuals. Although completing the COVID‐19 vaccination relatively lowered the risk of PE and DVT after COVID‐19, as compared with the unvaccinated participants, individuals with completion of the primary series still had a higher risk of PE after COVID‐19. A composite consideration of COVID‐19 and COVID‐19 vaccination with regards to its interaction may improve the effectiveness of management strategies and risk stratification against PE and DVT.

## 4 METHODS

### 4.1 Study population

A cohort database representative of the entire nation, provided by the Korea National Health Insurance Service (NHIS) and the KDCA to guarantee a comprehensive dataset, was employed in this study. Since January 1, 2020, the KDCA has been actively monitoring and gathering information on COVID‐19 diagnoses and vaccination within the Korean population. The database contains details such as the date of COVID‐19 diagnosis, vaccination dates, and the number of vaccine doses administered for each individual. The NHIS, in its commitment to ensuring equal access to high‐quality healthcare, provides mandatory insurance services to all Korean citizens. This facilitated the collection of extensive participant data, encompassing sociodemographic characteristics, medical history (including prescribed medications and treatment records), results of health screening examinations, lifestyle habits, serological aspects, and anthropometric measurements.^36^

In the course of this investigation, we combined two distinct databases supplied by KDCA and NHIS, compiling individuals who had a diagnosis of COVID‐19 from January, 2021 to March, 2022. The index date of follow‐up was determined as the day when an individual tested positive for COVID‐19 through real‐time PCR. To ensure comparability between COVID‐19 and non‐COVID‐19 groups, while minimizing heterogeneity, we employed a 1:10 exact matching based on the Charlson comorbidity index (CCI), household income, age, and sex. For individuals without COVID‐19, a fictitious index date was assigned after completing the matching process.

**FIGURE 3:**
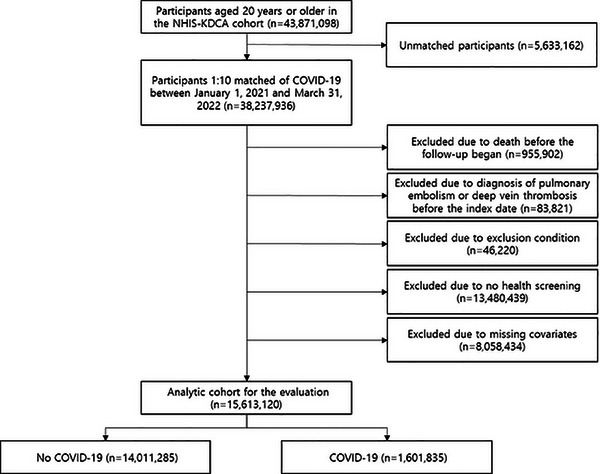
Flow diagram to derive the study population from South Korean population.

Prior to matching, there were 43,871,098 participants aged 20 years and over in the NHIS–KDCA cohort. The matching dropped 5,633,162 people, leaving 3,476,176 with COVID‐19 and 34,761,760 without. Those who died (*n* = 955,902) or had a history of PE or DVT (*n* = 83,821) prior to the first date of follow‐up investigation were excluded. Those who have had at least one day of hospitalization due to other thrombotic conditions before the index date, including cerebral venous thrombosis (International Classification of Diseases and Related Health Problems, 10th edition; ICD‐10: I676, I636, G08; *n* = 1,819), other venous embolism and thrombosis (ICD‐10: I822, I823, I828, I829; *n* = 7,366), disseminated intravascular coagulation (ICD‐10: D65; *n* = 430), and thrombocytopenia (ICD‐10: D693, D694, D695, D696; *n* = 36,605) were also excluded. Additionally, those who did not undergo health screening between 2018 and 2020 (*n* = 13,480,439), and those with missing covariates (*n* = 8,058,434) were also excluded. Finally, the analytic cohort included 1,601,835 COVID‐19 patients and 14,011,285 non‐COVID‐19 participants (Figure 3). This study adhered to the STROBE guidelines,^37^ with approval from the Seoul National University Hospital Institutional Review Board (No.: 2204‐126‐1319). In compliance with strict confidentiality standards and the anonymization of the database, the need for informed consents was exempted.

### 4.2 Follow‐up and outcome

Every participant was followed up from the date of their COVID‐19 diagnosis (or matched date) until the incident of outcomes, death, or June 30, 2022. To define cases of PE and DVT, we identified individuals who were hospitalized for at least one day with a primary diagnosis of PE (ICD‐10: I26) or DVT (ICD‐10: I801, I802, I803).

### 4.3 Key variables

The adjusted analyses took into account two continuous variables, including age (years) and body mass index (kg/m^2^), and several categorical variables as potential confounding factors: sex (male and female), household income (quartiles), hypertension (present and absent), diabetes (present and absent), dyslipidemia (present and absent), CCI (0, 1, and ≥2), cigarette smoking (never, past, and current), alcohol intake (at least once per week and none), and moderate and vigorous physical activity (0, 1−2, 3−4, and ≥5 times/week). The CCI was computed using the method outlined in another study.^38^ Hypertension, diabetes, and dyslipidemia were determined by cross‐referencing ICD‐10 codes I10, E10–E14, and E78, respectively, and reviewing prescription records of antihypertensive drugs, antidiabetic drugs, and lipid‐lowering drugs, respectively.

### 4.4 Statistical analysis

Continuous variables were presented in the form of mean and standard deviation, whereas categorical variables were expressed as count and percentage. This study utilized the Cox proportional hazards regression to evaluate the hazard ratio (HR) and 95% CIs for COVID‐19‐related PE and DVT. Three models were estimated: one crude and two adjusted models. The initial adjustment model incorporated age, sex, household income, diabetes, hypertension, dyslipidemia, body mass index, CCI, moderate and vigorous physical activity, smoking status, and alcohol intake as covariates. The second adjustment model employed a competing risk analysis model, with death due to any cause as competing events. To separate the effects of SARS‐CoV‐2 infection and COVID‐19 vaccination, the above analyses were conducted in a subgroup analysis format based on participants’ vaccination status, based on whether they had completed the primary series vaccination. The determination of each individual's status regarding COVID‐19 vaccination encompassed the administration of mRNA‐1273, BNT162b2, NVX‐CoV2373, ChAdOx1 nCoV‐19, and Ad26.COV2.S vaccines. Recipients who received Ad26.COV2.S vaccine (single dose) or ≥2 doses of other vaccines were considered completion of the primary series, whereas others were classified as unvaccinated.

Stratified analyses by age, sex, CCI, obesity (body mass index ≥25 and <25 kg/m^2^), hypertension, diabetes, and dyslipidemia were performed, with a goal of identifying specific population groups that may be at an elevated PE or DVT risk after SARS‐CoV‐2 infection.

Further analyses were conducted to explore the potential impact of COVID‐19 booster vaccinations, different virus variants, disease severity, and vaccine types on the risk of COVID‐19 related PE and DVT risk. Concerning the severity of COVID‐19, individuals under 60 years of age without underlying conditions (hypertension, diabetes, cardiovascular diseases, cancer, chronic obstructive pulmonary disease, liver disease, or sickle‐cell disorders) and without prescriptions for immunosuppressive medications, were classified into the mild‐low risk group. The mild‐high risk category comprised individuals aged 60 years or above, those with any of the specified underlying conditions, or those prescribed certain COVID‐19 medications (Molnupiraviar, Remdesivir, Ritonavir, Redanvimab) without needing advanced medical interventions (artificial ventilation, extracorporeal circulation, or oxygen therapy). The serious category included individuals prescribed COVID‐19‐specific medications (Olumiant, Actemra), without the need for advanced medical interventions (artificial ventilation, extracorporeal circulation, or oxygen therapy). Last, the severely serious category included individuals prescribed COVID‐19‐specific medications (Olumiant, Actemra), or those admitted to isolation rooms or intensive care units, and subjected to artificial ventilation, extracorporeal circulation, or oxygen therapy.

Individuals who did not contract SARS‐CoV‐2 were considered the reference group in each analysis. A significance level below 0.05 for a two‐tailed *p* value was considered as the threshold for significance. All data collection, data mining, and analyses were carried out utilizing SAS version 9.4 (SAS Institute Inc., Cary, NC, USA).

## AUTHOR CONTRIBUTIONS

H. J. K., S. J., and S. M. P. participated in the study conceptualization and study design. S. M. P. had full access to the data and ensured accuracy of the analyses. H. J. K. and S. J. carried out the statistical analyses. All authors participated in the result interpretation. H. J. K. and S. J. drafted the manuscript. All authors critically revised the manuscript for the clarity and intellectual contents. S. M. P. participated in the study supervision. All authors have read and approved the final manuscript.

## CONFLICT OF INTEREST STATEMENT

The authors declare no conflict of interest.

## ETHICS STATEMENT AND CONSENT TO PARTICIPATE

This study was approved by the Institutional Review Board of Seoul National University Hospital (No.: 2204‐126‐1319).

## CONSENT FOR PUBLICATION

In compliance with stringent confidentiality guidelines, the database was anonymized, thereby waiving the requirement for informed consent.

## Supporting information

Supporting Information

## Data Availability

The data supporting this study's findings are available from the NHIS but are not publicly accessible due to usage restrictions. However, they can be accessed from the authors upon reasonable request and with NHIS approval.
