# Supplementary material for: Risk of pulmonary embolism and deep vein thrombosis following COVID‐19: a nationwide cohort study
Source: MedComm (2020). 2024 Jul 14;5(7):e655. doi: 10.1002/mco2.655 (PMC11246596; doi:10.1002/mco2.655)
Supplement: Supplementary file 1 — Supporting Information [file MCO2-5-e655-s001.docx]

**INFORMATION**

**Title**: Risk of pulmonary embolism and deep vein thrombosis following COVID-19: a nationwide cohort study

**Authors**: Hye Jun Kim, MS, Seogsong Jeong, MD, PhD, Jihun Song, MS, Sun Jae Park, BS, Young Jun Park, MD, Yun Hwan Oh, MD, MS, Jaehun Jung, MD, PhD, Sang Min Park, MD, PhD, MPH

**Corresponding author**: Sang Min Park, MD, PhD, MPH. Department of Biomedical Sciences, Seoul National University. Department of Family Medicine, Seoul National University Hospital. 101 Daehak-ro, Jongno-gu, Seoul, South Korea. Phone: +82-2-2072-3331. Fax: +82-2-766-3276. E-mail: [smpark.snuh@gmail.com](mailto:smpark.snuh@gmail.com).

**Table S1**. Association of COVID-19 with pulmonary embolism based on COVID-19 vaccination doses

**Table S2**. Association of COVID-19 with deep vein thrombosis based on COVID-19 vaccination doses

**Table S3**. Association of COVID-19 with pulmonary embolism in the pre-omicron phase

**Table S4**. Association of COVID-19 with pulmonary embolism in the omicron phase

**Table S5**. Association of COVID-19 with deep vein thrombosis in the pre-omicron phase

**Table S6**. Association of COVID-19 with deep vein thrombosis in the omicron phase

**Table S7**. Association of the severity of COVID-19 with pulmonary embolism

**Table S8**. Association of the severity of COVID-19 with deep vein thrombosis

**Table S9**. Association of the types of COVID-19 vaccination with pulmonary embolism among participants without COVID-19

**Table S10**. Association of the types of COVID-19 vaccination with deep vein thrombosis among participants without COVID-19

**Table S1.** Association of COVID-19 with pulmonary embolism based on COVID-19 vaccination doses

|  | **No COVID-19**  (n=14,011,285) | **COVID-19**  (n=1,601,835) | ***P* value** | ***P* for interaction** |
| --- | --- | --- | --- | --- |
| Unvaccinated |  |  |  |  |
| Study population, n | 952,384 | 86,596 |  |  |
| Event, n (%) | 36 (0.00) | 22 (0.03) |  |  |
| Person-years | 503,268 | 49,441 |  |  |
| Median time-to-event, days | 90 (44-194) | 36 (26-86) |  |  |
| HR (95% CI) | 1.00 (reference) | 6.75 (3.97-11.47) | <0.001 | <0.001 |
| aHR (95% CI)^a^ | 1.00 (reference) | 6.25 (3.67-10.65) | <0.001 | <0.001 |
| aHR (95% CI)^b^ | 1.00 (reference) | 6.25 (3.67-10.66) | <0.001 | <0.001 |
| 2^nd^ dose vaccinated |  |  |  |  |
| Study population, n | 3,328,704 | 420,237 |  |  |
| Event, n (%) | 94 (0.00) | 23 (0.01) |  |  |
| Person-years | 1,139,115 | 140,515 |  |  |
| Median time-to-event, days | 62 (29-93) | 53 (25-78) |  |  |
| HR (95% CI) | 1.00 (reference) | 1.94 (1.23-3.06) | 0.004 |  |
| aHR (95% CI)^a^ | 1.00 (reference) | 2.01 (1.27-3.17) | 0.003 |  |
| aHR (95% CI)^b^ | 1.00 (reference) | 2.01 (1.28-3.16) | 0.003 |  |
| 3^rd^ dose or more vaccinated |  |  |  |  |
| Study population, n | 9,730,197 | 1,095,002 |  |  |
| Event, n (%) | 341 (0.00) | 51 (0.00) |  |  |
| Person-years | 2,907,865 | 325,367 |  |  |
| Median time-to-event, days | 50 (27-79) | 46 (27-79) |  |  |
| HR (95% CI) | 1.00 (reference) | 1.33 (0.99-1.79) | 0.058 |  |
| aHR (95% CI)^a^ | 1.00 (reference) | 1.31 (0.97-1.75) | 0.077 |  |
| aHR (95% CI)^b^ | 1.00 (reference) | 1.31 (0.97-1.75) | 0.075 |  |

HRs were evaluated using the Cox proportional hazards regression.

^a^Adjusted for age, sex, household income, Charlson comorbidity index, body mass index, hypertension, diabetes, dyslipidemia, moderate-to-vigorous physical activity, smoking, and alcohol consumption.

^b^Assessed using the competing risk model to calculate subdistribution hazard ratios after adjustments for variables in the model A with death as a competing risk.

Acronyms: COVID-19, coronavirus disease; HR, hazard ratio; CI, confidence interval; aHR, adjusted hazard ratio.

**Table S2.** Association of COVID-19 with deep vein thrombosis based on COVID-19 vaccination doses

|  | **No COVID-19**  (n=14,011,285) | **COVID-19**  (n=1,601,835) | ***P* value** | ***P* for interaction** |
| --- | --- | --- | --- | --- |
| Unvaccinated |  |  |  |  |
| Study population, n | 952,384 | 86,596 |  |  |
| Event, n (%) | 56 (0.01) | 16 (0.02) |  |  |
| Person-years | 503,247 | 49,439 |  |  |
| Median time-to-event, days | 105 (46-206) | 55 (33-203) |  |  |
| HR (95% CI) | 1.00 (reference) | 3.16 (1.81-5.50) | <0.001 | 0.020 |
| aHR (95% CI)^a^ | 1.00 (reference) | 3.05 (1.75-5.31) | <0.001 | 0.019 |
| aHR (95% CI)^b^ | 1.00 (reference) | 3.05 (1.75-5.29) | <0.001 | 0.028 |
| 2^nd^ dose vaccinated |  |  |  |  |
| Study population, n | 3,328,704 | 420,237 |  |  |
| Event, n (%) | 149 (0.00) | 20 (0.00) |  |  |
| Person-years | 1,139,098 | 140,517 |  |  |
| Median time-to-event, days | 65 (35-105) | 72 (31-121) |  |  |
| HR (95% CI) | 1.00 (reference) | 1.06 (0.67-1.70) | 0.795 |  |
| aHR (95% CI)^a^ | 1.00 (reference) | 1.11 (0.69-1.76) | 0.674 |  |
| aHR (95% CI)^b^ | 1.00 (reference) | 1.11 (0.69-1.77) | 0.674 |  |
| 3^rd^ dose or more vaccinated |  |  |  |  |
| Study population, n | 9,730,197 | 1,095,002 |  |  |
| Event, n (%) | 699 (0.01) | 91 (0.01) |  |  |
| Person-years | 2,907,801 | 325,361 |  |  |
| Median time-to-event, days | 61 (31-87) | 57 (28-81) |  |  |
| HR (95% CI) | 1.00 (reference) | 1.16 (0.93-1.44) | 0.190 |  |
| aHR (95% CI)^a^ | 1.00 (reference) | 1.16 (0.93-1.44) | 0.190 |  |
| aHR (95% CI)^b^ | 1.00 (reference) | 1.16 (0.93-1.44) | 0.190 |  |

HRs were evaluated using the Cox proportional hazards regression.

^a^Adjusted for age, sex, household income, Charlson comorbidity index, body mass index, hypertension, diabetes, dyslipidemia, moderate-to-vigorous physical activity, smoking, and alcohol consumption.

^b^Assessed using the competing risk model to calculate subdistribution hazard ratios after adjustments for variables in the model A with death as a competing risk.

Acronyms: COVID-19, coronavirus disease; HR, hazard ratio; CI, confidence interval; aHR, adjusted hazard ratio.

**Table S3.** Association of COVID-19 with pulmonary embolism in the pre-omicron phase

|  | **No COVID-19**  (n=14,011,285) | **COVID-19**  (n=76,805) | ***P* value** | ***P* for interaction** |
| --- | --- | --- | --- | --- |
| Unvaccinated |  |  |  |  |
| Study population, n | 952,384 | 36,203 |  |  |
| Event, n (%) | 36 (0.00) | 14 (0.04) |  |  |
| Person-years | 503,268 | 34,180 |  |  |
| Median time-to-event, days | 90 (44-194) | 38 (29-102) |  |  |
| HR (95% CI) | 1.00 (reference) | 10.28 (5.55-19.06) | <0.001 | 0.597 |
| aHR (95% CI)^a^ | 1.00 (reference) | 8.39 (4.51-15.63) | <0.001 | 0.458 |
| aHR (95% CI)^b^ | 1.00 (reference) | 8.39 (4.50-15.64) | <0.001 | 0.457 |
| Completion of the primary series |  |  |  |  |
| Study population, n | 13,058,901 | 40,602 |  |  |
| Event, n (%) | 435 (0.00) | 11 (0.03) |  |  |
| Person-years | 4,046,980 | 23,113 |  |  |
| Median time-to-event, days | 52 (27-82) | 42 (23-105) |  |  |
| HR (95% CI) | 1.00 (reference) | 8.17 (4.49-14.86) | <0.001 |  |
| aHR (95% CI)^a^ | 1.00 (reference) | 5.86 (3.22-10.66) | <0.001 |  |
| aHR (95% CI)^b^ | 1.00 (reference) | 5.86 (3.23-10.64) | <0.001 |  |

HRs were evaluated using the Cox proportional hazards regression.

^a^Adjusted for age, sex, household income, Charlson comorbidity index, body mass index, hypertension, diabetes, dyslipidemia, moderate-to-vigorous physical activity, smoking, and alcohol consumption.

^b^Assessed using the competing risk model to calculate subdistribution hazard ratios after adjustments for variables in the model A with death as a competing risk.

Acronyms: COVID-19, coronavirus disease; HR, hazard ratio; CI, confidence interval; aHR, adjusted hazard ratio.

**Table S4.** Association of COVID-19 with pulmonary embolism in the omicron phase

|  | **No COVID-19**  (n=14,011,285) | **COVID-19**  (n=1,525,030) | ***P* value** | ***P* for interaction** |
| --- | --- | --- | --- | --- |
| Unvaccinated |  |  |  |  |
| Study population, n | 952,384 | 50,393 |  |  |
| Event, n (%) | 36 (0.00) | 8 (0.00) |  |  |
| Person-years | 503,268 | 15,261 |  |  |
| Median time-to-event, days | 90 (44-194) | 26 (19-53) |  |  |
| HR (95% CI) | 1.00 (reference) | 4.21 (1.96-9.07) | <0.001 | 0.004 |
| aHR (95% CI)^a^ | 1.00 (reference) | 4.36 (2.03-9.40) | <0.001 | 0.003 |
| aHR (95% CI)^b^ | 1.00 (reference) | 4.36 (2.01-9.46) | <0.001 | 0.003 |
| Completion of the primary series |  |  |  |  |
| Study population, n | 13,058,901 | 1,474,637 |  |  |
| Event, n (%) | 435 (0.00) | 63 (0.00) |  |  |
| Person-years | 4,046,980 | 442,769 |  |  |
| Median time-to-event, days | 52 (27-82) | 49 (33-75) |  |  |
| HR (95% CI) | 1.00 (reference) | 1.28 (0.99-1.67) | 0.064 |  |
| aHR (95% CI)^a^ | 1.00 (reference) | 1.31 (1.00-1.70) | 0.047 |  |
| aHR (95% CI)^b^ | 1.00 (reference) | 1.31 (1.01-1.70) | 0.046 |  |

HRs were evaluated using the Cox proportional hazards regression.

^a^Adjusted for age, sex, household income, Charlson comorbidity index, body mass index, hypertension, diabetes, dyslipidemia, moderate-to-vigorous physical activity, smoking, and alcohol consumption.

^b^Assessed using the competing risk model to calculate subdistribution hazard ratios after adjustments for variables in the model A with death as a competing risk.

Acronyms: COVID-19, coronavirus disease; HR, hazard ratio; CI, confidence interval; aHR, adjusted hazard ratio.

**Table S5.** Association of COVID-19 with deep vein thrombosis in the pre-omicron phase

|  | **No COVID-19**  (n=14,011,285) | **COVID-19**  (n=76,805) | ***P* value** | ***P* for interaction** |
| --- | --- | --- | --- | --- |
| Unvaccinated |  |  |  |  |
| Study population, n | 952,384 | 36,203 |  |  |
| Event, n (%) | 56 (0.01) | 5 (0.01) |  |  |
| Person-years | 503,247 | 34,179 |  |  |
| Median time-to-event, days | 105 (46-206) | 146 (53-249) |  |  |
| HR (95% CI) | 1.00 (reference) | 2.36 (0.95-5.89) | 0.066 | 0.327 |
| aHR (95% CI)^a^ | 1.00 (reference) | 2.11 (0.84-5.28) | 0.112 | 0.290 |
| aHR (95% CI)^b^ | 1.00 (reference) | 2.11 (0.85-5.21) | 0.107 | 0.290 |
| Completion of the primary series |  |  |  |  |
| Study population, n | 13,058,901 | 40,602 |  |  |
| Event, n (%) | 848 (0.01) | 3 (0.01) |  |  |
| Person-years | 4,046,899 | 23,116 |  |  |
| Median time-to-event (case only) | 61 (32-89) | 135 (72-154) |  |  |
| HR (95% CI) | 1.00 (reference) | 1.14 (0.37-3.55) | 0.818 |  |
| aHR (95% CI)^a^ | 1.00 (reference) | 0.90 (0.29-2.79) | 0.851 |  |
| aHR (95% CI)^b^ | 1.00 (reference) | 0.90 (0.29-2.79) | 0.851 |  |

HRs were evaluated using the Cox proportional hazards regression.

^a^Adjusted for age, sex, household income, Charlson comorbidity index, body mass index, hypertension, diabetes, dyslipidemia, moderate-to-vigorous physical activity, smoking, and alcohol consumption.

^b^Assessed using the competing risk model to calculate subdistribution hazard ratios after adjustments for variables in the model A with death as a competing risk.

Acronyms: COVID-19, coronavirus disease; HR, hazard ratio; CI, confidence interval; aHR, adjusted hazard ratio.

**Table S6.** Association of COVID-19 with deep vein thrombosis in the omicron phase

|  | **No COVID-19**  (n=14,011,285) | **COVID-19**  (n=1,525,030) | ***P* value** | ***P* for interaction** |
| --- | --- | --- | --- | --- |
| Unvaccinated |  |  |  |  |
| Study population, n | 952,384 | 50,393 |  |  |
| Event, n (%) | 56 (0.01) | 11 (0.02) |  |  |
| Person-years | 503,247 | 15,260 |  |  |
| Median time-to-event, days | 105 (46-206) | 36 (29-49) |  |  |
| HR (95% CI) | 1.00 (reference) | 3.73 (1.95-7.11) | <0.001 | <0.001 |
| aHR (95% CI)^a^ | 1.00 (reference) | 3.86 (2.02-7.38) | <0.001 | <0.001 |
| aHR (95% CI)^b^ | 1.00 (reference) | 3.86 (2.02-7.38) | <0.001 | <0.001 |
| Completion of the primary series |  |  |  |  |
| Study population, n | 13,058,901 | 1,474,637 |  |  |
| Event, n (%) | 848 (0.01) | 108 (0.01) |  |  |
| Person-years | 4,046,899 | 442,762 |  |  |
| Median time-to-event, days | 61 (32-89) | 56 (27-80) |  |  |
| HR (95% CI) | 1.00 (reference) | 1.13 (0.92-1.38) | 0.238 |  |
| aHR (95% CI)^a^ | 1.00 (reference) | 1.16 (0.95-1.42) | 0.152 |  |
| aHR (95% CI)^b^ | 1.00 (reference) | 1.16 (0.95-1.42) | 0.152 |  |

HRs were evaluated using the Cox proportional hazards regression.

^a^Adjusted for age, sex, household income, Charlson comorbidity index, body mass index, hypertension, diabetes, dyslipidemia, moderate-to-vigorous physical activity, smoking, and alcohol consumption.

^b^Assessed using the competing risk model to calculate subdistribution hazard ratios after adjustments for variables in the model A with death as a competing risk.

Acronyms: COVID-19, coronavirus disease; HR, hazard ratio; CI, confidence interval; aHR, adjusted hazard ratio.

**Table S7.** Association of the severity of COVID-19 with pulmonary embolism

|  | **No COVID-19**  (n=14,011,285) | **COVID-19**  **(Mild)**  (n=1,579,735) | **COVID-19**  **(Serious-severely  serious)**  (n=21,847) | ***P* for trend** | ***P* for interaction** |
| --- | --- | --- | --- | --- | --- |
| Unvaccinated |  |  |  |  |  |
| Study population, n | 952,384 | 76,848 | 9,671 |  |  |
| Event, n (%) | 36 (0.00) | 13 (0.02) | 9 (0.09) |  |  |
| Person-years | 503,268 | 41,217 | 8,185 |  |  |
| HR (95% CI) | 1.00 (reference) | 4.49 (2.38-8.46)^***^ | 25.00 (12.03-51.85)^***^ | <0.001 | <0.001 |
| aHR (95% CI)^a^ | 1.00 (reference) | 4.37 (2.32-8.25)^***^ | 17.20 (8.20-35.97)^***^ | <0.001 | 0.001 |
| Completion of the primary series | |  |  |  |  |
| Study population, n | 13,058,901 | 1,502,887 | 12,176 |  |  |
| Event, n (%) | 435 (0.00) | 61 (0.00) | 13 (0.11) |  |  |
| Person-years | 4,046,980 | 460,750 | 5,086 |  |  |
| HR (95% CI) | 1.00 (reference) | 1.22 (0.93-1.59) | 32.57 (18.76-56.54)^***^ | <0.001 |  |
| aHR (95% CI)^a^ | 1.00 (reference) | 1.24 (0.95-1.62) | 15.15 (8.70-26.38)^***^ | <0.001 |  |

HRs were evaluated using the Cox proportional hazards regression.

^a^Adjusted for age, sex, household income, Charlson comorbidity index, body mass index, hypertension, diabetes, dyslipidemia, moderate-to-vigorous physical activity, smoking, and alcohol consumption.

Acronyms: COVID-19, coronavirus disease; HR, hazard ratio; CI, confidence interval; aHR, adjusted hazard ratio.

^***^*P* value<0.001.

**Table S8.** Association of the severity of COVID-19 with deep vein thrombosis

|  | **No COVID-19**  (n=14,011,285) | **COVID-19**  **(Mild)**  (n=1,579,735) | **COVID-19**  **(Serious-severely serious)**  (n=21,847) | ***P* for trend** | ***P* for interaction** |
| --- | --- | --- | --- | --- | --- |
| Unvaccinated |  |  |  |  |  |
| Study population, n | 952,384 | 76,848 | 9,671 |  |  |
| Event, n (%) | 56 (0.01) | 14 (0.02) | 2 (0.02) |  |  |
| Person-years | 503,247 | 41,216 | 8,185 |  |  |
| HR (95% CI) | 1.00 (reference) | 3.11 (1.73-5.58)^***^ | 3.57 (0.87-14.6) | <0.001 | 0.004 |
| aHR (95% CI)^a^ | 1.00 (reference) | 3.09 (1.72-5.55)^***^ | 2.79 (0.68-11.48) | <0.001 | 0.006 |
| Completion of the primary series | |  |  |  |  |
| Study population, n | 13,058,901 | 1,502,887 | 12,176 |  |  |
| Event, n (%) | 848 (0.01) | 107 (0.01) | 3 (0.02) |  |  |
| Person-years | 4,046,899 | 460,743 | 5,090 |  |  |
| HR (95% CI) | 1.00 (reference) | 1.10 (0.90-1.34) | 3.86 (1.24-11.98)^*^ | 0.186 |  |
| aHR (95% CI)^a^ | 1.00 (reference) | 1.12 (0.92-1.37) | 2.20 (0.71-6.83) | 0.160 |  |

HRs were evaluated using the Cox proportional hazards regression.

^a^Adjusted for age, sex, household income, Charlson comorbidity index, body mass index, hypertension, diabetes, dyslipidemia, moderate-to-vigorous physical activity, smoking, and alcohol consumption.

Acronyms: COVID-19, coronavirus disease; HR, hazard ratio; CI, confidence interval; aHR, adjusted hazard ratio.

^*^*P* value<0.05. ^***^*P* value<0.001.

**Table S9.** Association of the types of COVID-19 vaccination with pulmonary embolism among participants without COVID-19

|  | **Unvaccinated** | **Completion of the primary series** | **First and second dose vaccination** | |
| --- | --- | --- | --- | --- |
|  |  |  | **mRNA-mRNA** | **Viral-Viral** |
| Study population, n | 952,384 | 13,058,901 | 8,776,756 | 3,056,545 |
| Event, n (%) | 36 (0.00) | 435 (0.00) | 244 (0.00) | 174 (0.01) |
| Person-years | 503,268 |  | 2,707,881 | 954,887 |
| aHR (95% CI)^a^ | 1.00 (reference) | 0.62 (0.44-0.87)^**^ | 0.65 (0.46-0.93)^*^ | 0.60 (0.41-0.86)^**^ |
| aHR (95% CI)^a^ |  |  | 1.00 (reference) | 0.91 (0.74-1.11) |

HRs were evaluated using the Cox proportional hazards regression.

^a^Adjusted for age, sex, household income, Charlson comorbidity index, body mass index, hypertension, diabetes, dyslipidemia, smoking, alcohol consumption, and moderate-to-vigorous physical activity.

Acronyms: COVID-19, coronavirus disease; mRNA-mRNA, administration of mRNA vaccine for the 1^st^ and 2^nd^ dose of COVID-19 vaccine; Viral-viral, administration of viral vector vaccine for the 1^st^ and 2^nd^ dose of COVID-19 vaccine; aHR, adjusted hazard ratio; CI, confidence interval; HR, hazard ratio.

mRNA vaccine includes Pfizer-BioNTech (BNT162b2) and Moderna (mRNA-1273) vaccine.

Viral vector vaccine includes AstraZeneca (ChAdOx1 nCov-19) and Janssen (AD26.COV2-S) vaccine.

^*^*P* value<0.05. ^**^*P* value<0.01.

**Table S10.** Association of the types of COVID-19 vaccination with deep vein thrombosis among participants without COVID-19

|  | **Unvaccinated** | **Completion of the primary series** | **First and second dose vaccination** | |
| --- | --- | --- | --- | --- |
|  |  |  | **mRNA-mRNA** | **Viral-Viral** |
| Study population, n | 952,384 | 13,058,901 | 8,776,756 | 3,056,545 |
| Event, n (%) | 56 (0.01) | 848 (0.01) | 484 (0.01) | 310 (0.01) |
| Person-years | 503,247 |  | 2,707,837 | 954,857 |
| aHR (95% CI)^a^ | 1.00 (reference) | 0.84 (0.64-1.10) | 0.86 (0.65-1.13) | 0.79 (0.59-1.05) |
| aHR (95% CI)^a^ |  |  | 1.00 (reference) | 0.91 (0.78-1.06) |

HRs were evaluated using the Cox proportional hazards regression.

^a^Adjusted for age, sex, household income, Charlson comorbidity index, body mass index, hypertension, diabetes, dyslipidemia, moderate-to-vigorous physical activity, smoking, and alcohol consumption.

Acronyms: COVID-19, coronavirus disease; mRNA-mRNA, administration of mRNA vaccine for the 1^st^ and 2^nd^ dose of COVID-19 vaccine; Viral-viral, administration of viral vector vaccine for the 1^st^ and 2^nd^ dose of COVID-19 vaccine; aHR, adjusted hazard ratio; CI, confidence interval; HR, hazard ratio.

mRNA vaccine includes Pfizer-BioNTech (BNT162b2) and Moderna (mRNA-1273) vaccine.

Viral vector vaccine includes AstraZeneca (ChAdOx1 nCov-19) and Janssen (AD26.COV2-S) vaccine.
